# Step-by-step Peritoneal Bladder Flap Bunching (PBFB) technique: an innovative approach following lymph node dissection in robotic radical prostatectomy

**DOI:** 10.1590/S1677-5538.IBJU.2024.0278

**Published:** 2024-05-31

**Authors:** Ahmed Gamal, Marcio Covas Moschovas, Abdel Rahman Jaber, Shady Saikali, Sumeet Reddy, Ela Patel, Evan Patel, Travis Rogers, Vipul Patel

**Affiliations:** 1 AdventHealth Global Robotics Institute USA AdventHealth Global Robotics Institute, Celebration, USA; 2 University of Central Florida Orlando USA University of Central Florida - UCF, Orlando, USA; 3 Stanford University Palo Alto California USA Stanford University, Palo Alto, California, USA

## Abstract

**Introduction:**

Robot-assisted radical prostatectomy (RARP) has become a popular surgical approach for localized prostate cancer due to its favorable oncological and functional outcomes, as well as lower morbidity. In cases of intermediate- and high-risk prostate cancer, bilateral pelvic lymphadenectomy (PLND) is recommended as an adjunct to RARP ( 1 – 3 ). Despite its benefits, PLND can lead to surgical complications, with postoperative lymphocele formation being the most common. Most postoperative lymphoceles are clinically insignificant with variable incidence, reaching up to 60% of cases 4. However, a small percentage of patients 2-8% may experience symptomatic lymphoceles (SL), which can cause significant morbidity ( 4 , 5 ).

**Surgical technique:**

We perform our RARP technique with our standard approach in all patients ( 6 ). After vesicourethral anastomosis a modified PF created to prevent symptomatic lymphocele. We start by suturing the peritoneal fold on the right side, medially to the vas deferens, followed by a similar stitch on the left side to approximate the edges in the midline. A running suture bunches the bladder peritoneum from both sides, passing through the pubic bone periosteum to secure it in place ( 7 ). This approach keeps the lateral pelvic gutters open for lymphatic drainage, while allowing fluid drainage from the true pelvis into the abdomen. A pelvic ultrasound was done for all patients at 6 weeks post operative, and additional clinical follow-up was carried out at 3 months following surgery.

**Considerations:**

We have demonstrated a modified technique of peritoneal flap (PBFB) with an initial decrease in postoperative symptomatic lymphoceles, the technique is feasible, safe, does not add significant morbidity, and does not require a learning curve.

Available at: http://www.intbrazjurol.com.br/video-section/20240278_Patel_et_al

**Int Braz J Urol. 2024; 50 (Video #9): 657-8**
